# Undifferentiated nasopharyngeal cancer extending to maxillary sinus: a case report

**DOI:** 10.11604/pamj.2020.36.276.24766

**Published:** 2020-08-13

**Authors:** Mohamed Beghdad, Amine Mkhatri, Yassine Harmoumi, Meriem Doumiri, Sami Rouadi, Reda Abada, Mohamed Roubal, El Benna Naima, Mohamed Mahtar

**Affiliations:** 1 ENT Department, 20 August Hospital, Ibn Rochd Teaching Hospital, Casablanca, Morocco,; 2 Radiology Department, Ibn Rochd Teaching Hospital, Casablanca, Morocco

**Keywords:** Undifferentiated nasopharyngeal cancer, maxillary sinus, computed tomography

## Abstract

Undifferentiated nasopharyngeal cancer of the cavum (UCNT) is the most frequent neoplasm of the nasopharynx, having a close relationship with exposure to Epstein-Barr virus. It has a high potential for locoregional or distant invasion which are the cause of some treatment failures. The extension to the maxillary sinus is rarely described. We report here the case of a 38-year-old patient with headaches associated with epistaxis, left otalgia and facial pain. Examination by anterior rhinoscopy objectively revealed a polylobed ulcerating mass. Otoscopic examination revealed a left seromucous otitis media. Computed tomography showed a voluminous tumour process in the infra temporal fossa and nasopharynx with significant locoregional extension particularly in the maxillary sinus. Pathological examination revealed an UCNT of the cavum and the patient was classified as T4N2M0. The patient received chemoradiotherapy, with wide irradiation of the cervical lymph node areas. The deep localization of the cancer of the cavum, which is difficult to examine, requires a diagnostic and extension work-up, both endoscopic and radiological, which is an important step in the diagnostic and therapeutic management.

## Introduction

Carcinomas of the cavum, in particular undifferentiated, are the most frequent tumours of the nasopharynx [1]. They have a high potential for locoregional or distant invasion and are the cause of certain therapeutic failures [2]. It is therefore essential in the extension assessment to analyse in detail this crossroads area and the multiple possible extensions, in particular to the foramens of the skull base and to the deep spaces, which allows good diagnosis and therapeutic management and avoids recurrences [3]. We report in this paper the case of a nasopharyngeal cancer with important locoregional extension particularly in the maxillary sinus.

## Patient and observation

**Figure 1:**
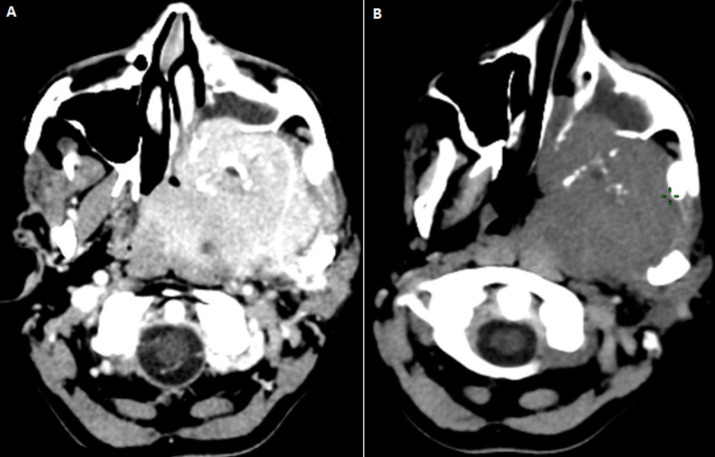
A) cervical CT scan in axial sections without and with injection showing the hypodense tumour of the cavum; B) enhancing intensely after injection of contrast agent. It occupies the left infra temporal fossa with invasion of the nasal fossa and the homolateral maxillary sinus

**Figure 2:**
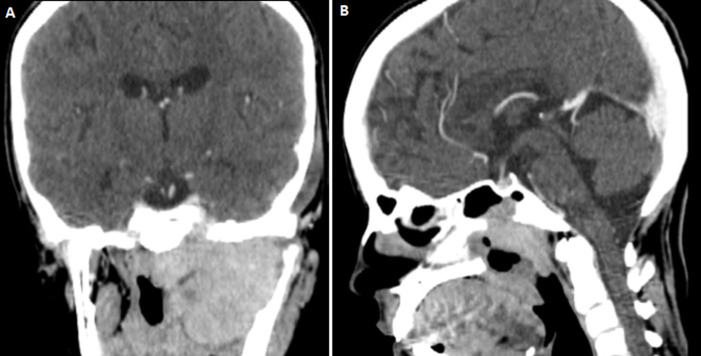
A) cervical CT scan with sagittal reconstruction showing the endocranial extension of the tumour through the carotid canal; B) with the lysis of the floor of the sphenoidal sinus

A young patient of 38 years of age who smokes, presented at the otolaryngology consultation for headaches associated with epistaxis, left otalgia and facial pain. The clinical examination revealed a nasopharyngeal polylobal ulcerated mass with left seromucous otitis. A cervicofacial computed tomography (CT) showed a large tumour process in the left infra temporal fossa, poorly limited, hypodense, enhancing after injection and measuring approximately 62x50mm. It extends anteriorly to the left maxillary sinus with lysis of its walls and internally the pterygoid region, the nasal fossae, the posterior ethmoid cells and the homolateral choana (Figure 1). Lateralally, it comes into contact with the left jugal soft tissues deforming the hemi-mandible, which is the site of a periosteal reaction on its ascending branch, with lysis of the cortex in some areas. It lyses the bony palate with opposing soft part extension, at the top it invades the middle level of the skull base, through the carotid canal and the sphenoidal sinus (Figure 2). It pushes back the left lateral wall of the nasopharynx and oropharynx with invasion of the parapharyngeal fat. Bilateral laterocervical lymph nodes have also been found, the largest of which measures 10mm in left sector II. The pathological examination revealed an UCNT. The tumor was classified as T4N2M0. The patient received chemoradiotherapy, with wide irradiation of the cervical lymph node areas.

## Discussion

The UCNT is particular among other head and neck neoplasms considering its epidemiology, histopathology and treatment [4,5]. Its geographical distribution is extremely unbalanced. More than 70% of new cases are in East and Southeast Asia, with an age-standardised rate of 3.0 per 100,000 in China to 0.4 per 100,000 in populations that are mainly white [6,7]. Except host genetics, Epstein-Barr virus is perhaps the most common causal agent of UCNT. Other potential risk factors are family history of UCNT, active and passive tobacco smoking, consumption of preserved foods and alcohol and oral hygiene [8-12]. Patients with UCNT can suffer from rhinological symptoms (epistaxis, nasal obstruction and discharge), otological symptoms (tinittus and deafness), neurological symptoms (headaches, diplopia and facial pain) and neck mass [5]. UCNT is an aggressive neoplasm that can spread frequently in the paranasal sinuses. The most common site of invasion is the sphenoid sinus (21%), followed by maxillary sinus (11.2%) and the ethmoid sinus (4.9%) [13]. According to Li Tian, the invasion of the paranasal sinus is an independent negative prognostic factor for overall survival, distant metastasis and local relapse in patients with UCNT [13]. Cross-sectional imaging has improved the effectiveness of treatment for UCNT. Magnetic resonance imaging (MRI) is better than CT for assessing superficial and deep nasopharyngeal soft tissue and for differentiating tumor from soft tissue [14]. However, the MRI is less effective for displaying bone details. Consequently, CT should be undertaken whenever the status is the skull base cannot be correctly assessed with the MRI. Concurrent chemoradiotherapy remains the most efficacious treatment for UCNT [8]. Intensity modulated radiotherapy demonstrated an improved therapeutic ratio compared with two-dimensional radiotherapy and decrease in most late toxicities and noncancer death. However, this modality of treatment remains insufficient in terms of distant control [15,16]. Further studies on the role of Epstein-Barr virus (EBV) latent proteins could help to indentify other novel treatment targets given the close association between EBV and UCNT.

## Conclusion

Maxillary sinus invasion in patients with UCNT is relatively uncommon. This invasion is defined as T3 disease according to AJCC staging system 8^th^ edition. This invasion is an independent negative prognostic factor for UCNT.
